# High Temperature Epoxy Foam: Optimization of Process Parameters

**DOI:** 10.3390/polym8060215

**Published:** 2016-06-07

**Authors:** Samira El Gazzani, Valérie Nassiet, Jean-Pierre Habas, Christian Freydier, Aline Hilleshein

**Affiliations:** 1 Université de Toulouse, INPT, Laboratoire Génie de Production, Ecole Nationale d'Ingénieurs de Tarbes, 47 Avenue d’Azereix, B.P. 1629, 65016 Tarbes cedex, France; samira.elgazzani@enit.fr (S.E.G.); alinehilleshein@hotmail.com (A.H.); 2 Roxel France, N151, 18570 Le Subdray, France; c.freydier@roxelgroup.com; 3 Institut Charles Gerhardt, Equipe Ingénierie et Architectures Macromoléculaires, UMR CNRS 5253, CC 1702, Université de Montpellier, Place Eugène Bataillon, 34095 Montpellier, France; jean-pierre.habas@univ-montp2.fr

**Keywords:** expandable microsphere, rigid foam, TETM trifunctional epoxy, thermomechanical analysis

## Abstract

For many years, reduction of fuel consumption has been a major aim in terms of both costs and environmental concerns. One option is to reduce the weight of fuel consumers. For this purpose, the use of a lightweight material based on rigid foams is a relevant choice. This paper deals with a new high temperature epoxy expanded material as substitution of phenolic resin, classified as potentially mutagenic by European directive Reach. The optimization of thermoset foam depends on two major parameters, the reticulation process and the expansion of the foaming agent. Controlling these two phenomena can lead to a fully expanded and cured material. The rheological behavior of epoxy resin is studied and gel time is determined at various temperatures. The expansion of foaming agent is investigated by thermomechanical analysis. Results are correlated and compared with samples foamed in the same temperature conditions. The ideal foaming/gelation temperature is then determined. The second part of this research concerns the optimization of curing cycle of a high temperature trifunctional epoxy resin. A two-step curing cycle was defined by considering the influence of different curing schedules on the glass transition temperature of the material. The final foamed material has a glass transition temperature of 270 °C.

## 1. Introduction

Polymer–fiber composite materials are widely used in many applications as they combine a low weight with satisfying mechanical properties. A decrease in weight is a recurrent requirement in industry, particularly in the aerospace field where fuel cost has a significant impact. Producing foamed materials is a way to achieve mass reduction.

There are different methods to produce foam: decomposition of chemical substance with gas formation, under heating [1,2,3,4]; bubble creation by vaporization of a liquid [5,6,7,8,9,10]; gas introduction in the resin under high-pressure follow-up with expansion when pressure drops [11,12,13]; and mechanical beating [14,15]. The low boiling temperature liquids used for the expansion by vaporization are usually hydrofluorocarbon, which is problematic because they contribute to global warming. Other methods allow lightening a structure, for example by filling a matrix with microballoons to produce syntactic foam [16,17,18], or with water-soluble crystals in a net immersed in water so that holes supplant the crystals [19,20]. However, these processes do not involve expansion, so they are not relevant for our application.

In the case of thermoset foams, the foaming and the crosslinking reaction have to take place simultaneously. Sometimes, the blowing agent is involved in the crosslinking reaction. For example, the reaction between isocyanate and water, which produce CO_2_, is a well-known method used in the manufacture of polyurethane foams. Li *et al.* [21] and Basso *et al.* [22,23] explored the addition of isocyanate in the manufacture of tannin foams. Dogan and Kusefoglu [24] studied the reaction between malonic acid and epoxidized soybean oil. Malonic acid reacts with epoxy groups on the one hand and produces CO_2_ by decarboxylation on the other hand, which acts as a blowing agent leading to *in situ* foaming of the polymer. Stefani *et al.* [25] used a polysiloxane as blowing agent to produce epoxy foam. They postulate that the amino hardener reacts with the polysiloxane, leading to dihydrogen production. Indeed, the amino hardener has a dual function: crosslinking agent and gas co-producer. These two reactions follow distinct kinetics, which make the crosslinking/foaming process difficult to control. Then, the use of a foaming agent independent of a crosslinking reaction would be preferable. For example, sodium bicarbonate is a harmless substance that can be used as a blowing agent since it decomposes during heating, releasing CO_2_ and H_2_O. It was used in various studies with epoxy resins [1,26]. Najib *et al.* used it as a foaming agent in natural rubber [27].

Expandable microsphere is a non-traditional physical blowing agent initially patented by Morehouse in 1971 [28]. The expandable microspheres are prepared by a suspension-type polymerization of droplets of a mixture of monomers and blowing agents. This leads to a microsphere of thermoplastic copolymer encapsulating a volatile hydrocarbon liquid. Expansion occurs because the thermoplastic shell softens, while the encapsulated hydrocarbon gasifies, increasing the internal pressure in the particle. The copolymer is made of three different monomers assumed to have a specific role: the one with the lowest glass transition temperature (*T*_g_) offers initial softening as the liquid starts to vaporize; the one with middle *T*_g_ allows the expansion of the microspheres; and the one with highest *T*_g_ lends a relative stiffness. The particles expand as the hydrocarbon vapor pressure overcomes the yield strength of the polymer shell [29]. In order to contain the gas, a monomer with gas-barrier properties such as acrylonitrile (AN) or methacrylonitrile (MAN) is generally used as a polymerizable monomer [30]. There is a large range of products, made of different copolymers and hydrocarbons, leading to various temperatures of expansion. They are commercially available under the trade name of Expancel® [31]. This has been used for different purposes like car protection production [32] and for the manufacture of an expanded composite material named Roxalte®.

Roxalte® is developed by Roxel, a company dedicated to propulsion systems, and is of increasing interest to the aerospace industry. This material is made from three major components: phenolic resin, a basalt fiber mat, and an expandable microsphere [33].

The phenolic resin is a resole type, composed of a hydroxymethyl phenol, phenol, and formaldehyde solution. The high concentration of aromatic cycles after curing provides excellent thermal properties at high temperatures. Some tests were performed on the phenolic resin and a glass transition temperature of 380 °C was found [34].

Anyway, phenol and formaldehyde are both hazardous molecules, classified as potentially mutagenic and carcinogenic, respectfully, by European directive Reach. The substitution of this resin is then required. Epoxy resin has been selected as a relevant choice due to its good mechanical properties, resistance to corrosive liquids and environments, resistance to moisture absorption, and durability. The main criterion of selection of epoxy bi-components was to obtain a high glass transition temperature (above 250 °C). The link between the chemical structure of the material and the *T*_g_ was studied by various authors [35,36]. The *T*_g_ has been found to increase with the degree of crosslinks and chain rigidity of the polymer network [37]. The degree of crosslinking is inversely proportional to the average molecular weight between crosslinks (*M*c). Then molecules with a high reactive group ratio should enhance the *T*_g_. On the other hand, the chain rigidity is greater for an aromatic cycle than for aliphatic ones [38]. The link between the presence of an aromatic cycle and the high *T*_g_ of the structure is illustrated in Van Krevelen’s study [39]. In order to reach a high *T*_g_, the triglycidyl ether of tris(4-hydroxyphenyl)methane (TETM), a trifunctional epoxy with high aromatic ratio, has been chosen. Indeed, the high energy chemical bonds of the aromatic cycle are responsible for the properties’ stability at high temperature. Because it is an aromatic amine, diaminodiphenylsulfone (DDS) has been selected as curing agent. Moreover, TETM is known to have superior thermal oxidative stability over other types of epoxy resins [40]. Aronhime and Guillham estimated theoretically the glass transition temperature of TETM/DDS formulation at about 352 °C [41].

For the reasons previously exposed, the substitution of phenolic resin in the Roxalte was the first purpose of this study. Subsequently, different foaming processes were considered. Starting with the blowing agent used in the Roxalte, *i.e.* expandable microspheres, seemed logical. Indeed, as far as we know, the association of the preselected epoxy formulation with these microspheres has not been studied. Further developments on other foaming processes with different foaming agents will be the aim of another paper.

Wang *et al.* [42] produced epoxy/expandable microsphere foams in two steps. The epoxy was diglycidyl-ether of bisphenol-A. A precure step was first done on the epoxy/curing agent/blowing agent formulation in order to increase the viscosity by starting the polymerization process. The foaming process occurred during the second step where the formulation was put under a higher temperature. This process intended to improve the cell size distribution in the foam. In our study, we propose manufacturing the foam in one step only, by tuning the microsphere’s foaming kinetic to the resin gelation kinetic. The crosslinking reaction and the expansion of microspheres take place simultaneously. Obtaining an expanded material depends on two distinct phenomena, the foaming agent expansion on one hand and the resin gelation on the other hand. These two phenomena are independent of each other but both depend on time and temperature.

First, we propose a complete physicochemical study of expandable microspheres, in order to understand their behavior. In particular, the expansion is studied by thermomechanical analysis and correlated to the gel time of the epoxy formulation with the aim of identifying the ideal process temperature and producing the optimized foamed material. The optimization of the epoxy formulation curing cycle has been realized to achieve the manufacture of enhanced expanded foam.

## 2. Materials and Methods

### 2.1. Materials

**Figure 1:**
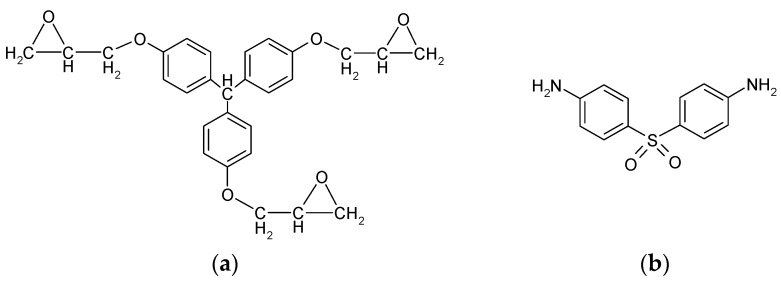
Chemical structures of (**a**) TETM and (**b**) DDS.

The triglycidyl ether of tris(4-hydroxyphenyl)methane (TETM) and the diaminodiphenylsulfone (DDS) were kindly offered by Huntsman Advanced Materials (Basel, Switzerland). Their commercial names are Tactix 742 and Aradur 976, respectively. The foaming agents from the Expancel® range were supplied by Rossow Industries (Gennevilliers, France). The Expancel® 950 DU 80 was selected (Particule size 18–24 μm; Density ≤12 kg/m^3^). The microsphere copolymer is made of acrylonitrile, methylmetacrylate, and methacrylonitrile and the volatile liquid inside is an isooctane–isopentane mix. Its technical data sheet also reveals the presence of magnesium hydroxide. The chemical structures of TETM and DDS are presented in Figure 1.

### 2.2. Preparation of Epoxy Resin

**Figure 2:**
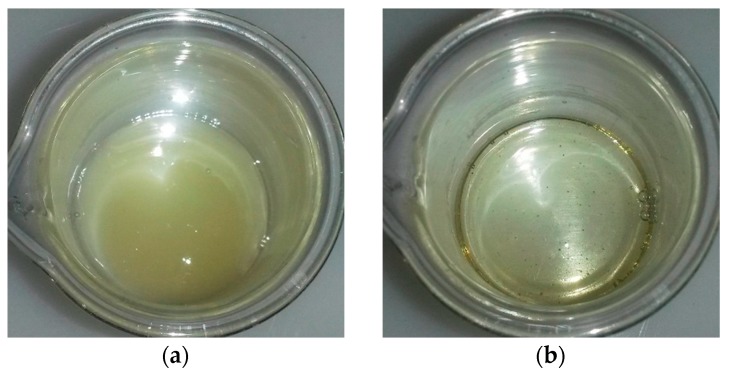
Picture of TETM/DDS mixed at (**a**) 100 °C and (**b**) 180 °C.

The epoxy resin formulation was prepared in stoichiometric proportion: the stoichiometric ratio, denoted as r, is defined by the ratio between the number of amine and epoxy group and has to be equal to 1. Then, the mass proportions were 71% of TETM for 29% of DDS. The melting temperature of DDS is 178 °C. As evidence, the TETM was first heated at 180 °C and the DDS was added and mixed under mechanical stirring until apparent homogeneity. Indeed, we can see in Figure 2 the difference between the TETM/DDS formulations mixed at 100 and 180 °C. At 100 °C, the viscosity is small enough to allow mixing but the DDS is not melted, leading to a low homogeneity, compared to the mix realized at 180 °C.

The mix is cooled at ambient temperature before starting any analyses.

### 2.3. Preparation of Epoxy Foam

To prepare the epoxy foam, TETM was first mixed into DDS at 180 °C, as above. Then the temperature was lowered to 100 °C and the microspheres were added and mixed under mechanical stirring again until apparent uniformity. First experiments were useful to evaluate the necessary quantity of blowing agent. Finally, the mixture was poured into an aluminum pot and cured in an oven at constant temperature.

### 2.4. Scientific Characterization

All differential scanning calorimetry (DSC Q 200 from TA Instruments, New Castle, DE, USA) analyses were performed under inert atmosphere (N_2_) using an aluminum crucible with a calibrated hole and an empty crucible as reference.

First, the temperature domain of curing reaction of epoxy system was determined by DSC. The reactive formulation was prepared as mentioned before and a small sample of about 5 mg was removed for testing.

To understand the expandable microspheres behavior, DSC experiments were performed in dynamic mode, on usual microspheres and on compressed microspheres. The compressed microspheres were obtained using a manual hydraulic press (Specac, 15 tons). A few grams of microspheres were introduced into the press and a 9-ton load was applied. This allows for throwing the liquid hydrocarbons out. The DSC analyses were performed between −20 and 400 °C, at a temperature ramp of 3 °C/min. To be free from liquid hydrocarbon evaporation and to focus on determination of the glass transition temperature, DSC analyses were carried out on another sample as follows: a sample of compressed microspheres has been heated from −50 to 200 °C, at a rate of 10 °C/min, then cooled before a second temperature scan at 10 °C/min to *T*_g_ determination. The first heating allows for evacuating the residual volatile liquid and the second one allows the *T*_g_ measurement. Thermogravimetric analyses (TGA-50 Shimadzu, Kyoto, Japan) were achieved in order to get the degradation temperature. Experiments were performed between 25 and 500 °C, at temperature ramps of 1, 5, 10, and 20 °C/min, under air atmosphere.

The study of expansion of expandable microspheres was performed by thermomechanical analysis (TMA 7 from Perkin Elmer, Waltham, MA, USA). This instrument is used to measure the dimensional variation of a sample under constant strain, as a function of time or temperature. A low strain is applied by a probe on the sample to maintain the contact between the probe and the sample, and the position of the probe is registered. The tests were done in isothermal mode, chosen to be in the appropriate temperature range of the curing reaction. A temperature ramp of 30 °C/min was applied until the isothermal temperature was reached. The strain applied was 10 mN.

The determination of the gel time of the epoxy system was realized by viscosimetric experiment using a dynamic rheometer (MCR 302 from Anton Paar, Graz, Austria) equipped with a cup-plate geometry, in which the diameter of the upper plate is much smaller than the diameter of the cup (25 and 41 mm), in order to prevent side effects and slippage. The gel time is measured at the divergence of viscosity and strongly depends on the temperature used in the kinetic. So the experiment was performed in isothermal mode, chosen to be in the appropriate temperature range of the curing reaction, in steady state with a shear rate of 20 s^−1^. For these experiments, the reactive formulations were prepared at a temperature of 180 °C. The mixture was then poured into the dedicated cup in the instrument and the test started when the temperature was reached.

Scanning electronic microscopy (SEM) was used to study the morphology of the foam. SEM was done using model ZEISS EVO HD15 (Jena, Germany). The variable pressure (VP) imaging was carried out at 30 Pa. There was no need to coat non-conductive materials prior to analysis. The incident electron beam was kept at 10 kV.

The study of optimization of the curing cycle of the epoxy system was performed by DSC and thermomechanical analysis by using a dynamic rheometer equipped with rectangular torsion geometry. First, epoxy formulations were subjected to different curing times (30, 40, 50, 60, 120, and 180 min) at the same temperature. Then, around 6 mg of each material were used for DSC analysis. Tests were performed at a heating rate of 5 °C/min, from 25 to 400 °C. Samples for thermomechanical analysis were prepared as follows. The epoxy formulation was mixed, poured into an aluminum mold, and cured in an oven under a determined cycle. The samples stayed in the oven for slow cooling. The dimensions of the final samples were 2 mm × 8 mm × 45 mm. Tests were performed with a temperature ramp of 3 °C/min, from 25 to 350 °C, with a deformation of 0.1% and an angular frequency of 1 rad/s.

Thermomechanical analyses using a dynamic rheometer equipped with rectangular torsion geometry were also performed on epoxy foam. The foamed material was cut to form a parallelepipedal sample (3 mm × 12 mm × 45 mm). Tests were performed with a temperature ramp of 3 °C/min, with an angular frequency of 1 rad/s and a deformation of 0.05%.

## 3. Results and Discussion

### 3.1. Preliminary Study of the Epoxy Formulation and Expandable Microspheres Characterization

**Figure 3:**
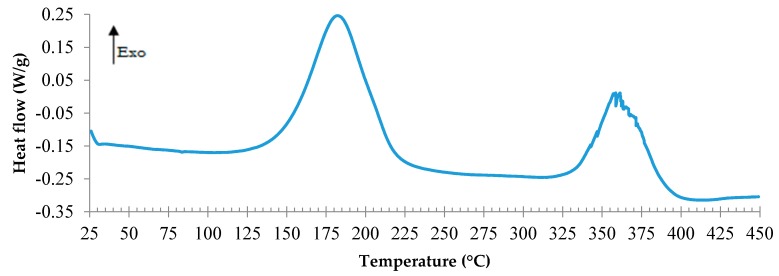
DSC scan of TETM/DDS formulation performed under nitrogen at a heating rate of 3 °C/min.

First, the epoxy formulation was studied by DSC in order to determine the temperature range of the curing reaction. The DSC thermogram exposed in Figure 3 shows a first exothermic peak corresponding to the curing reaction and a second one, noisy and repeatable, probably due to the degradation. It is presumed that the exothermic peak of degradation is due to the condensation of polyaromatic species by radical carbonization, as was postulated by Rose *et al.* [43]. According to the DSC curve, this process starts around 330 °C. It can be observed that the first exothermic peak is included between 130 and 240 °C, for a heating rate of 3 °C/min.

Since the reaction starts below 180 °C, the question was asked about the mixing temperature of the formulation. In order to know whether the gelation started during the mixing, the gel times were determined at different temperatures for two mixes: one mixed at 100 °C, and the second at 180 °C. Results are gathered in Table 1. The gel times prove the difference between the two mixes. Anyway, if the mix is realized at 100 °C, we may have some concerns about DDS precipitation as it is not well mixed into the epoxy prepolymer (Figure 2). Thus, we have chosen to perform the mixing at 180 °C, aware that the gelation is only slight. However the gelation did not continue when the microspheres were added and mixed at 100 °C, as the curing reaction only starts at 130 °C (see Figure 3).

Thermochemical characterizations (DSC and TGA) were performed on compressed and uncompressed microspheres to understand their behavior.

**Figure 4:**
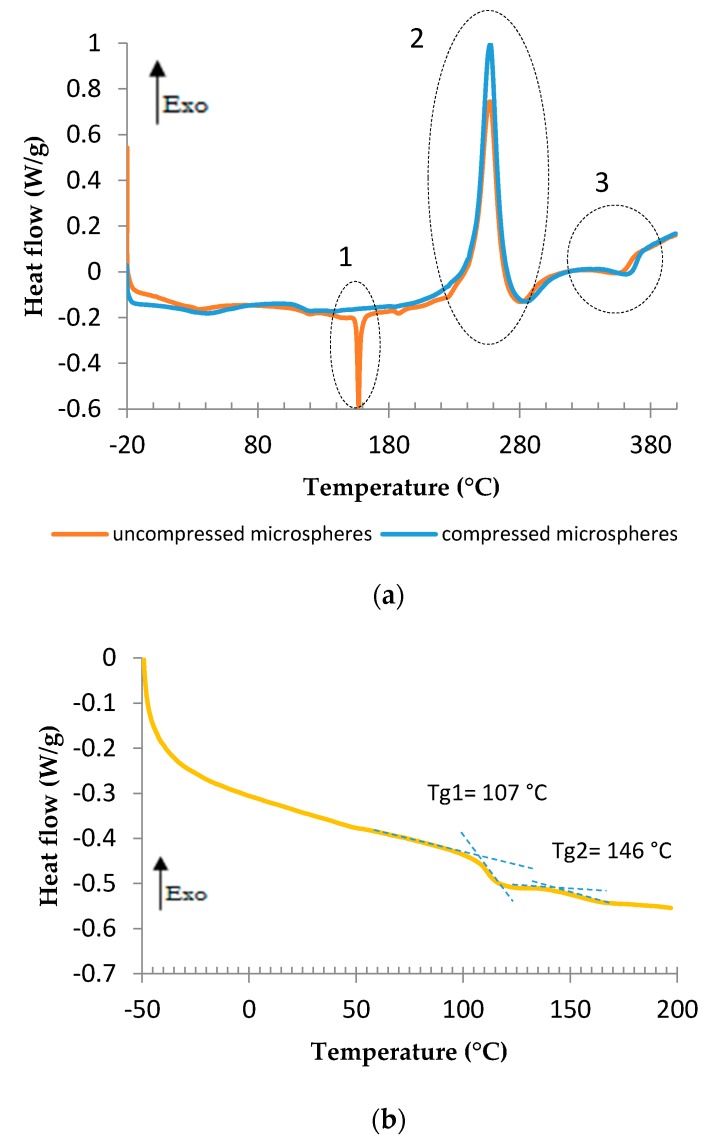
DSC analyses of compressed and uncompressed microspheres at 3 °/min, with the three major reactions encircled (**a**) and DSC analysis performed on compressed microspheres (second heating) for the determination of glass transitions at 10 °C/min (**b**).

The DSC analyses performed on compressed and uncompressed microspheres are presented in Figure 4a. There are three major reactions. The first one, measured at 160 °C, appears for uncompressed microspheres only. This is certainly due to the hydrocarbon liquid vaporization. According to the technical data sheet, there are two hydrocarbons, isopentane and isooctane. Their vaporization temperatures are 28 and 99 °C, respectively, at atmospheric pressure. However, when the microspheres are unexpanded, the internal pressure in this confined space is higher than in atmospheric conditions, leading to a higher temperature of vaporization [44]. A pressure drop is possible when the microspheres expands, leading to the evaporation of the condensed blowing agent. The vaporization occurred here at around 160 °C, which is therefore the temperature of starting expansion. This vaporization should correspond to isooctane. Therefore, the vaporization of isopentane is not noticeable. It was supposed that the proportion of this hydrocarbon was too low to be clearly detected by DSC. Figure 4b exposes the analyses performed on compressed microspheres to reveal the glass transition temperature. A major *T*_g_ appears around 107 °C and a secondary one around 146 °C. There are various *T*_g_ values reported in the literature for the components of the copolymer capsule (PMMA, PAN, PMAN) [45,46], because the *T*_g_ is depending on molecular weight. We do not know the molecular weight of the copolymers so it is difficult to conclude which copolymer is related to these *T*_g_. However, we assumed that the first glass transition (*T*_g_1) offers initial softening to reduce local pressure in the inner part of cell wall; the second *T*_g_ (*T*_g_2) leads to the softening of the outer part of the cell wall, allowing the hydrocarbon vaporization due to drop in pressure. One of the three copolymers is not associated with any *T*_g_; we assume that its role is to ensure relative stiffness. The proximity of these two *T*_g_ values made us think of a *T*_g_ gradient, in which the third copolymer *T*_g_ should be contained.

**Figure 5:**
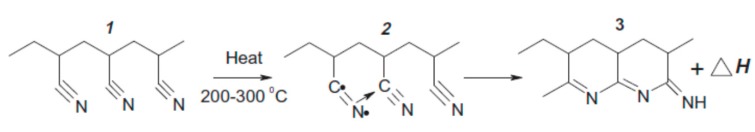
Cyclization reaction in polyacrylonitrile [47].

**Figure 6:**
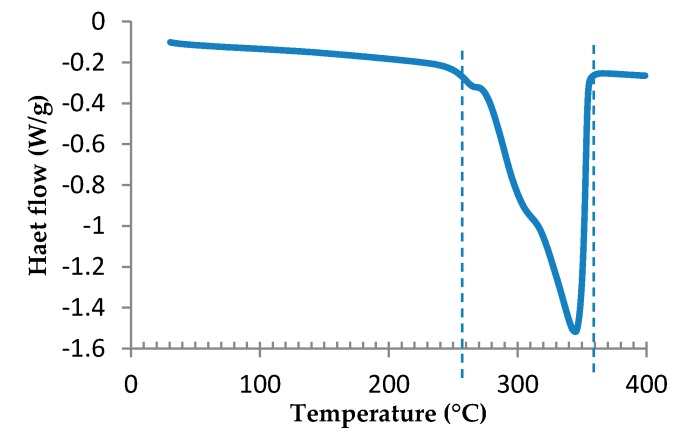
DSC analysis of magnesium hydroxide, 3 °C/min.

The second and third major reactions appear for both compressed and uncompressed microspheres. It is thus assumed that they are related to the capsule of the microspheres. The exothermic peak, between 220 and 290 °C, is due to the cyclization reaction in polyacrylonitrile and polymetacrylonitrile [47,48], exposed in Figure 5. The third reaction is an endothermic peak between 320 and 380 °C. It is attributed to the decomposition of magnesium hydroxide to form magnesium oxide and water, as illustrated in Figure 6, where the thermogram of magnesium hydroxide is represented. We can see that the endothermic peak is between 260 and 360 °C.

**Figure 7:**
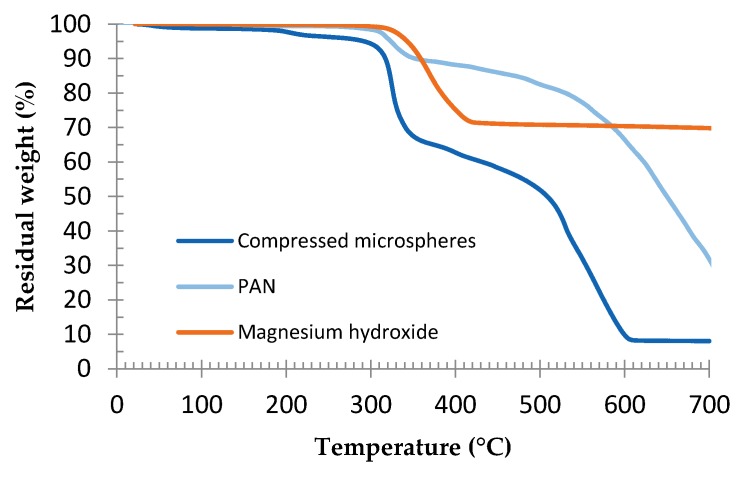
Comparison of TGA of compressed microspheres, PAN (polyacrylonitrile) and magnesium hydroxide, at 10 °C/min, under air. For PAN analysis, see Korobeinyk *et al.* [47].

In order to confirm the phenomena responsible for the weight loss, the TGA curves, performed at 10 °C/min, of compressed microspheres, PAN, and magnesium hydroxide were compared (Figure 7). PAN analysis is reported in a study by Korobeinyk *et al.* [47]. It is observed that the mass loss due to the PAN cyclization reaction matches the major microspheres’ weight loss. A similar mechanism is observed for PMAN [48]. We can also see that magnesium hydroxide degradation occurs in the same temperature range.

**Figure 8:**
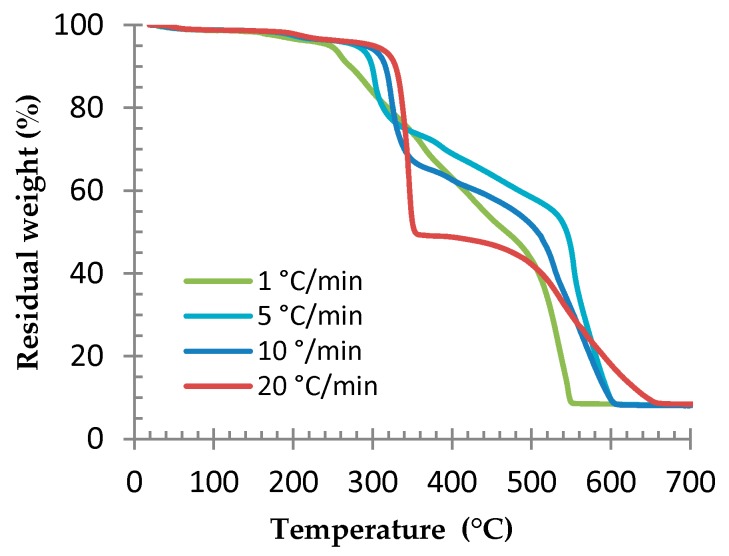
TGA of compressed microspheres at various temperature ramps, under air.

Thermogravimetric analyses were performed at various heating rates on compressed microspheres in order to determine the degradation temperature. Results are reported in Figure 8. The degradation temperature and polymer degradation depend on the temperature ramp. Moreover, when the heating is performed slowly, the degradation phenomenon happens gradually. On the contrary, when heating is performed quickly, the main degradation kinetics are graphically clear, which explains the shape differences between the curves.

**Figure 9:**
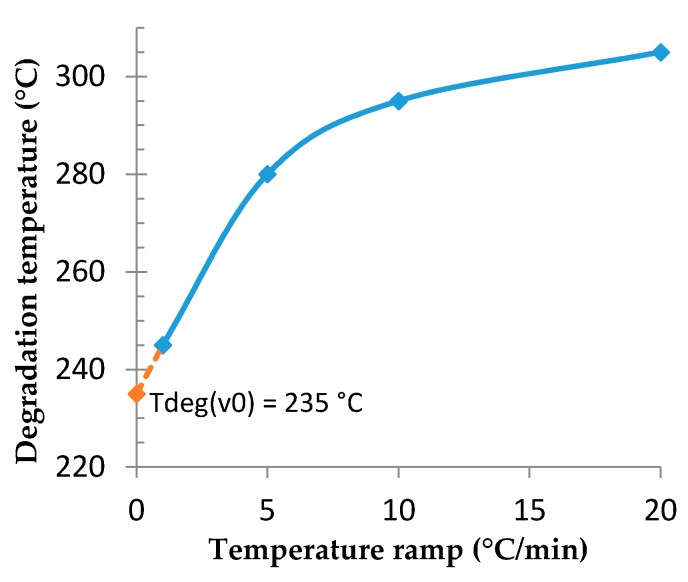
Influence of temperature ramp on the degradation temperature of compressed microspheres, by TGA, under air.

For every curve, a small decrease of about 2%–3% appears around 200 °C; this is attributed to the vaporization of residual hydrocarbon liquid. Then, for every curve the degradation temperature is measured at a mass loss of 5% to take into account the polymer’s degradations. The curve of the degradation temperature in function of the temperature ramp is drawn and the degradation temperature at a very low heating rate (close to 0 °C/min) is determined by extrapolation. As is shown in Figure 9, this value is around 235 °C. The 8.5% residual mass corresponds to the magnesium oxide formation, which occurs between 260 and 360 °C according to the heating rate and resists temperatures up to 700 °C, all the other carbon species being transformed to CO_2_.

### 3.2. Determination of the Optimal Foaming Temperature

In our process, we propose to manufacture the foam in only one step. Then we need to determine the temperature that leads to equilibrium between the microsphere’s foaming kinetic and the resin gelation kinetic. First, the gel time of the epoxy system is determined at different temperatures, after which the microsphere’s foaming is studied.

**Figure 10:**
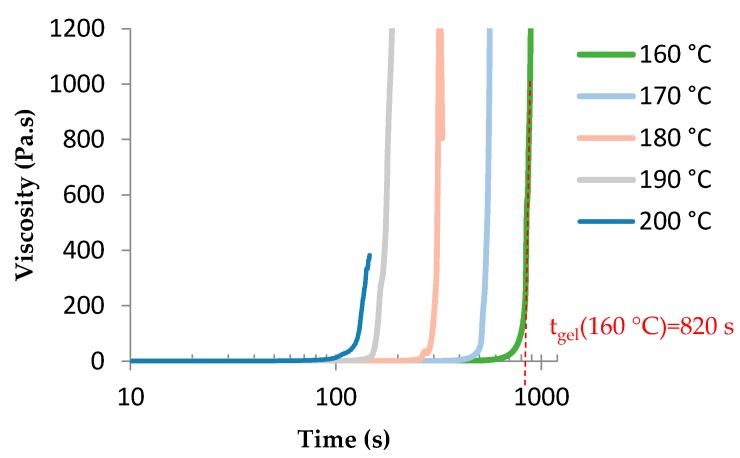
Determination of the gel time of TETM/DDS formulation at various temperatures.

Gelation time is measured at the divergence of viscosity and obviously depended on the temperature, as shown in Figure 10.

**Figure 11:**
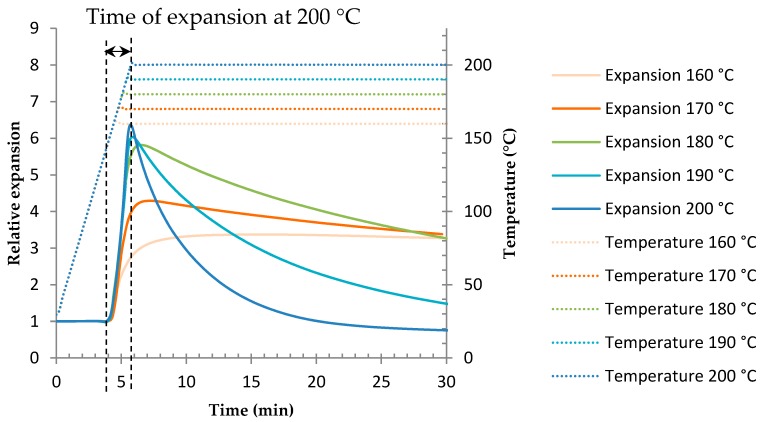
TMA of expandable microspheres Expancel® 950 DU 80 at various temperatures.

Microsphere expansion study was performed by TMA at isothermal temperatures, from 160 °C to 200 °C. Results of analysis are given in Figure 11. The relative expansion is the ratio of the sample thickness to the initial sample thickness. For each temperature, this deformation of expansion reaches a maximum value then drops. There are two phases: expansion and shrinkage. The deformation of expansion is higher and is happening faster when the temperature increases. This is because of the higher foaming temperature generated by a higher pressure, which stretches the cell wall even more, as was reported by other authors [27,42]. At 180, 190, and 200 °C the expansion is quite important compared to at temperatures of 160 and 170 °C. The shrinkage phase is even faster when the temperature is high.

It has to be noted that the expansion starts during the temperature ramp after 4 min, that is to say at 150 °C. This temperature corresponds with the *T*_g_2 (see Section 3.1) responsible for the expansion. The time of expansion corresponds to the expansion phase: from *t* = 4 min to the maximum value of the curve, as shown in Figure 11 for the temperature of 200 °C.

**Figure 12:**
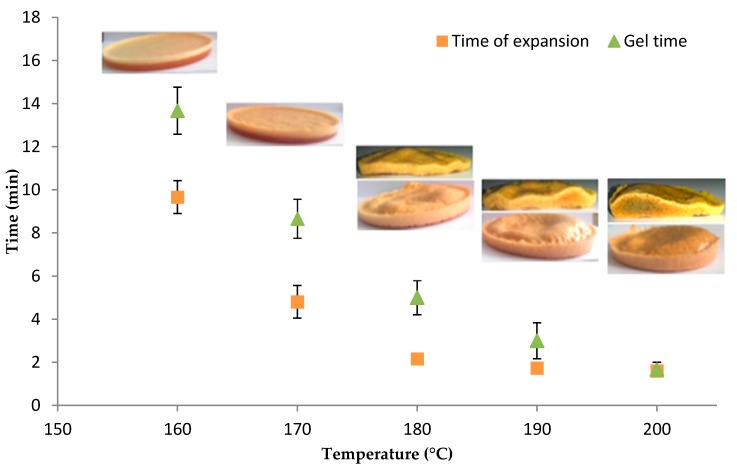
Microspheres TMA and epoxy system gel time analysis: correlation of results.

Results from the TMA and epoxy system gel time analysis are gathered in Figure 12. TETM/DDS/Microspheres foam samples were manufactured as described in Section 2 and cured at a constant temperature from 160 to 200 °C, with the aim of comparing with the previous analysis. The pictures are given in Figure 12, associated with each temperature.

Between 160 and 180 °C, the gel time is quite high compared to the time of expansion. At 160 and 170 °C the pictures show that expansion is very limited and a phase separation can be observed: the bubbles are lighter than the resin and go up to the surface. This phenomenon can occur because the resin gelation occurs later on. At 180 °C, the picture shows a great expansion, without phase separation. Indeed, as was shown in Figure 11, at this temperature the expansion is relatively important. Moreover, even if the gelation occurs late, the shrinkage kinetic is quite slow so the bubble size decreases slowly.

Between 190 and 200 °C, the gel time is close to the time of expansion. The expansion is important and faster; there is no phase separation. At these temperatures the shrinkage kinetic is fast, as is shown in Figure 11, which confirms that the gelation has to be close to the time of expansion to avoid the shrinkage of the foam. Furthermore, a progressive darkening is observed inside the sample at 190 and 200 °C. This coloration is due to the exothermicity of the curing reaction so the temperature in the sample is greater than the isothermal temperature in the oven. Therefore it is reasonable to think that an oxidative reaction occurred in these samples. The cause of this oxidation is explained later in the text.

The density data for these samples were measured by using a pycnometer and are gathered in Table 2. The density decreases with the temperature, which confirms that the expansion is more important at 190 and 200 °C than at 180 °C. However, a better compromise would be a temperature of 180 °C, because it allies a great expansion with a slow shrinkage and avoids thermal degradation.

**Figure 13:**
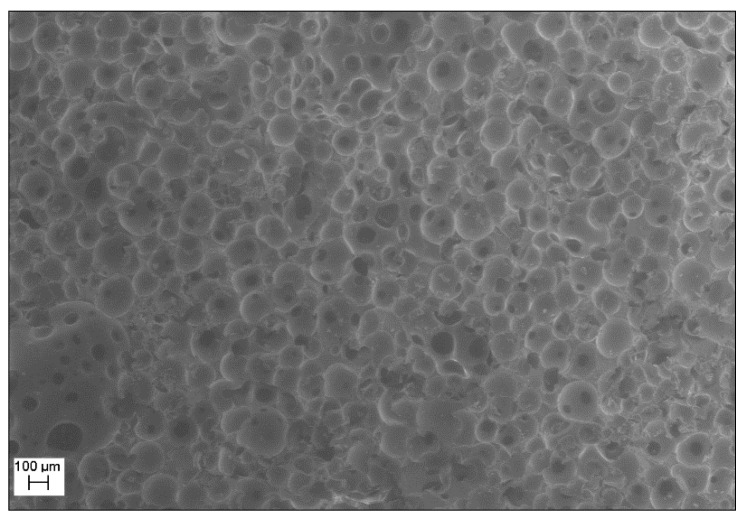
SEM micrograph of the foam sample (magnification 30x).

A SEM (scanning electronic microscopy) micrograph of the epoxy formulation/expandable microsphere foamed at 180 °C is given in Figure 13. The cell size distribution is homogeneous, which is a sign of good synchronization between the occurrence of the gelation and the time of expansion [42].

In order to observe the behavior of different grades of expandable microspheres, three additional expandable microspheres grades were selected and tested at 180 °C (920 WUF 40, 920 DU 40, 980 DUX 120). They were chosen according to their expansion temperature, which was around 180 °C. Referring to the supplier data sheet, these four grades are made of the same copolymer: acrylonitrile–methylmetacrylate–methacrylonitrile. However, the hydrocarbons inside the shell are not the same for all. For instance, 920 WUF 40 and 920 DU 40 contain isopentane, 980 DUX 120 contains isooctane, and 950 DU 80 contains an isopentane–isooctane mix. As was explained in Section 3.1, expansion is possible only if the shell is softened enough. Then the hydrocarbons are allowed to expand. Knowing the nature of the copolymer is not sufficient because the *T*_g_ is dependent on the molecular weight of each copolymer. So the nature of the gas inside the shell as well as the mass of each copolymer is needed to predict the temperature of expansion.

Four epoxy formulation/expandable microspheres mixes were prepared. The epoxy formulations were prepared as before, in an aluminum crucible, at 180 °C. The temperature was dropped to 100 °C, at which time the microspheres were added and mixed until apparent homogeneity. The four mixtures had exactly the same weight and a proportion of 5% of microspheres. Then, the crucibles were placed in an oven at 180 °C, for 30 min.

**Figure 14:**
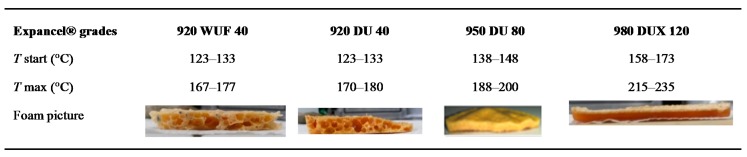
Expansion temperature data of microspheres and pictures of expandable microspheres/epoxy formulation foams cured at 180 °C.

The pictures of the cured mixes with the four different expandable microspheres are presented in Figure 14. *T*_start_ corresponds to the temperature when the foaming begins and *T*_max_ corresponds to the temperature when the microsphere volume is maximal. These values are issued from the technical data sheet. The samples were cut down the middle into two parts; we can see the 5 cm length profiles. The samples containing the 920 WUF 40 and 920 DU 40 microspheres show average expansion and bubbles of heterogeneous and large dimensions. This is certainly due to coalescence. As the bubbles grow, adjacent ones tend to coalesce in order to reduce the total free energy by lowering the surface area of cells [49,50,51]. This is happening here because the thermoplastic capsule collapsed due to the high temperature while the crosslinking reaction was not really consequent. Indeed, it has been demonstrated by various authors that too low a viscosity increases the probability of coalescence [50,52].

The sample containing the 950 DU 80 microspheres presents greater expansion and bubbles of homogeneous and microscopic dimensions.

The sample containing the 980 DUX 120 microspheres shows a low expansion, on the surface only. Referring to producer data, this grade needs higher temperature to expand than previous ones. The temperature in the core is not high enough to produce a whole expansion compared to the surface because of the weak thermal conductivity of epoxy resins.

### 3.3. TETM/DDS Cure Cycle Optimization

**Figure 15:**
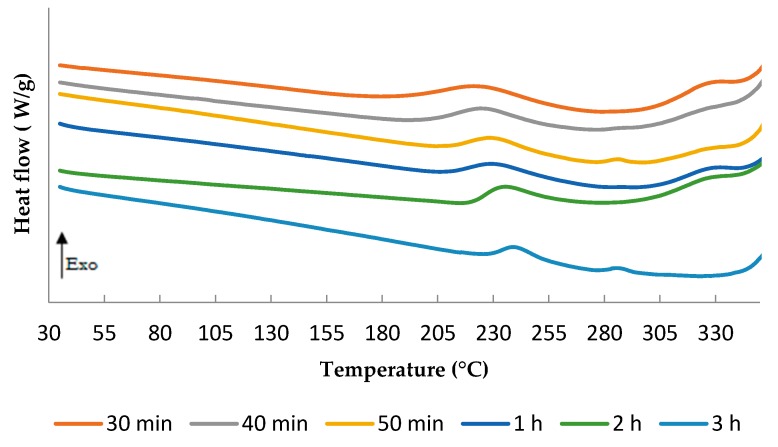
DSC scan of TETM/DDS formulations after various times of cure at 180 °C, performed under nitrogen with a heating rate of 5 °C/min.

Based on the previous study, a first temperature T_1_ = 180 C was set for the curing process of the TETM/DDS system. DSC analyses were conducted as described before. Results are presented in Figure 15.

An exothermic peak is remained around 240 °C for each time of cure. The longer the curing lasts, the lower the remaining peak height. However, even after 3 h at 180 °C, the cure is not completed. The *T*_g_ depends on the degree of cure reaction [53]. Then the system needs to be fully cured to increase the *T*_g_.

A post-curing temperature *T*_2_ = 240 °C was then added to the cycle. To determine the optimal cure thermal cycle, six curing cycles were prepared and tested by thermomechanical analysis using a dynamic rheometer equipped with rectangular torsion geometry:

C_1_: 1 h at180 °C + 1 h at 240 °C
C_2_: 1 h at 180 °C + 2 h at 240 °C
C_3_: 2 h at 180 °C + 1 h at 240 °C
C_4_: 2 h at 180 °C + 2 h at 240 °C
C_5_: 3 h at 180 °C + 1 h at 240 °C
C_6_: 3 h at 180 °C + 2 h at 240 °C

The temperature ramp between the two stages was 3 °C/min. DSC analyses of each sample after the two steps curing cycles were performed and no remaining peak was observed.

**Figure 16:**
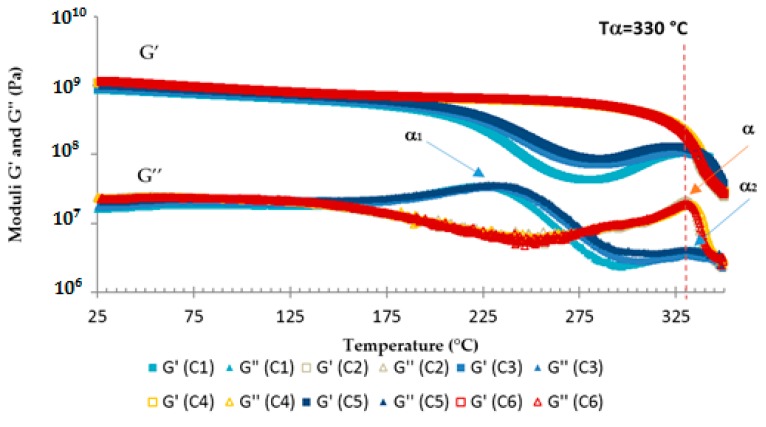
Rheological analyses of TETM/DDS formulations cured according to various cure cycles.

Results from rectangular torsion tests, performed at an angular frequency of 1 rad/s, are gathered in Figure 16. The samples that went through a second stage of 1 h at 240 °C (C_1_, C_3_, and C_5_) are similar, likewise the samples that had a second stage of 2 h at 240 °C (C_2_, C_4_, and C_6_). Two alpha relaxations, a rheological signature of glass transition temperature, are observed at the peak on the *G*’’ curves of C_1_, C_3_, and C_5_ samples, α_1_ at 230 °C, and α_2_ at 330 °C. Indeed, *G*’’ is sensitive to the long-range molecular motion occurring during the α relaxation [54]. α_1_ indicates molecular movements, which confirms that the curing process is not over and that more time for more energy is needed around this temperature. Evolution in the polymerization reaction is occurring during this analysis. The macromolecules’ conformation allows for the reaction of chemical entities (α_1_). As the heating is slow during the test, the molecules have the time to react, leading to α_2_, which actually is at the same temperature as α. Then, this test is allowed to complete the curing reaction for samples that went through a second stage of only 1 h at 240 °C (C_1_, C_3_, and C_5_). The second stage of 2 h at 240 °C is then needed to complete the curing process. However, a first stage of 1 h at 180 °C is enough since there is no difference when increasing the time of the first stage from 1 h to 3 h (C_2_, C_4_, and C_6_). Finally, the optimized cure cycle is 1 h at 180 °C and 2 h at 240 °C (C_2_). The alpha (α) temperature, evaluated at the peak on the *G*’’ curve, is then equal to 330 ± 2 °C.

**Figure 17:**
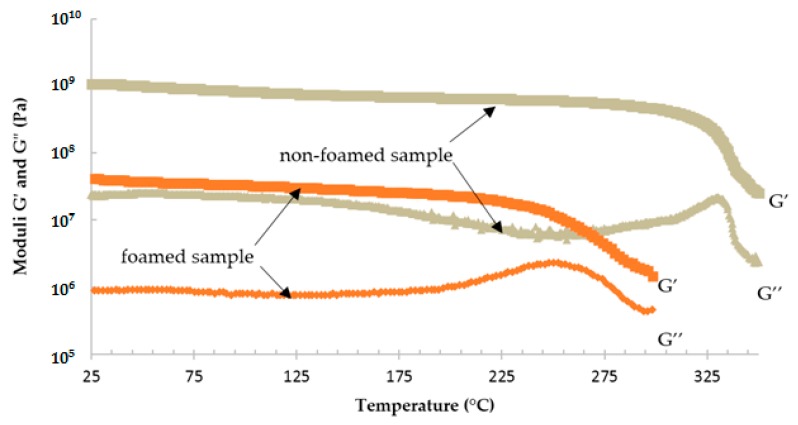
Comparison of rheological analyses of foamed and non-foamed sample, cured according to C_2_ cycle.

Epoxy foam was realized as described in Section 2. The mixture was cured in an oven, where the cycle C_2_ was applied. Parallelepipedal samples were cut from the foamed material and rectangular torsion analyses were performed. Results are given in Figure 17, with the curves of the non-foamed sample previously obtained. The moduli values of the foam are logically lower because of air presence. The *T*_α_ of the foam is also less important. Indeed, the endothermic reaction due to the vaporization of the liquid during the expansion of the microspheres consumes a part of the heat released during the exothermic cure reaction of the TETM/DDS system.

**Figure 18:**
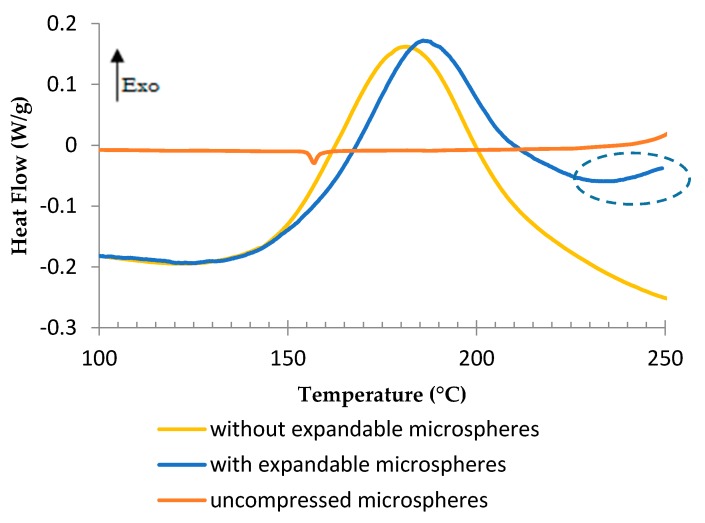
DSC analyses of compressed microspheres and TETM/DDS formulation with and without expandable microspheres, under air, at a heating rate of 3 °C/min.

The DSC analyses in Figure 18 allow us to compare the thermograms of TETM/DDS formulation, one containing 5% of expandable microspheres and one without microspheres, with the thermogram of expandable microspheres only. The value of heat flow for microspheres was reduced to the weight of hydrocarbons contained in the epoxy formulation with expandable microspheres, as they are responsible for the endothermic reaction, or 1% of the epoxy formulation (20% of hydrocarbons in 5% of microspheres). There is a small interval of 5 °C between the peaks on the thermograms of the formulation without and with microspheres, corresponding to 15 min. This temperature shift is explained by the endothermic peak of vaporization, which delays the curing reaction. As the expansion happens during the first stage of the curing process (at 180 °C), it was postulated that more time at this temperature was needed to complete the curing reaction of the foam. At temperatures over 230 °C, the increased heat flow encircled in the thermogram of epoxy formulation with microspheres is related to a degradation, which is discussed later.

**Figure 19:**
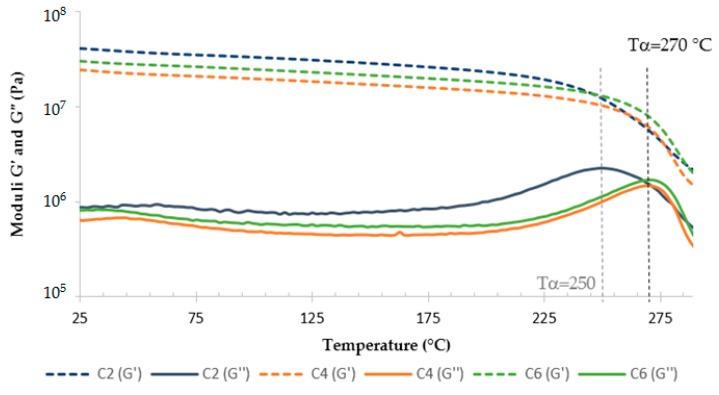
Rheological analyses of TETM/DDS/expandable microspheres foam cured according to various cure cycles, with a heating rate of 3 °C/min.

Then two other cycles were tested for 2 h at 180 °C + 2 h at 240 °C, and 3h at 180 °C + 2 h at 240 °C (C_4_ and C_6_). Results are given in Figure 19. Applying the cure cycle C_2_ allows for reaching 250 ± 2 °C as the glass transition temperature. Increasing the time of the first stage of 1 h allows for increasing the glass transition temperature by about 20 °C, leading to a *T*_α_ of 270 ± 2 °C. However, a first stage of 3 h at 180 °C is not needed.

**Figure 20:**
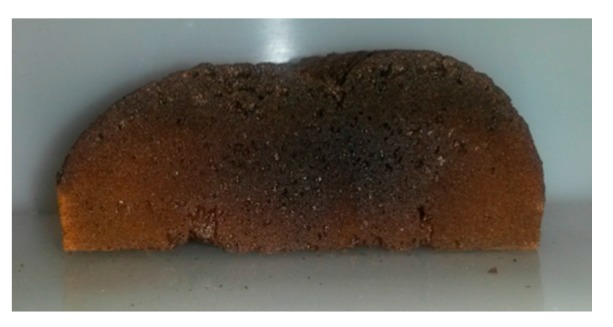
Cross-sectional picture of TETM/DDS/expandable microspheres foam, cured according to the C_2_ cycle (length = 7 cm).

**Figure 21:**
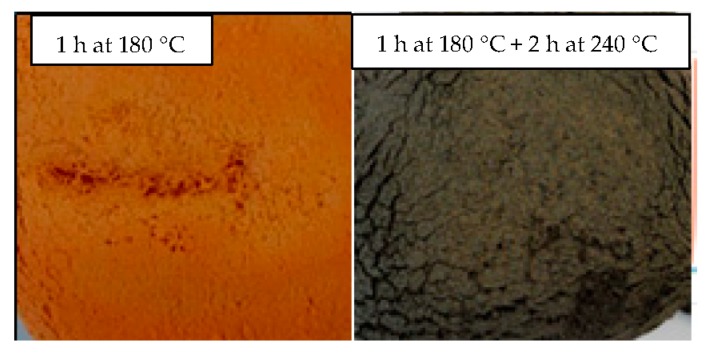
Pictures of samples cured without a post-cure (**left**) and with a post-cure (**right**) at 240 °C.

The foams presented a dark coloration, as shown in Figure 20. We assume that this coloration is due to the degradation of expandable microspheres and particularly to the polymerization of sequences of adjacent nitrile groups [55], in the cyclization reaction occurring in polyacrylonitrile and polymethacryclonitrile [47,48]. Indeed, this darkening occurred during the post-curing at 240 °C, as is highlighted in Figure 21.

**Figure 22:**
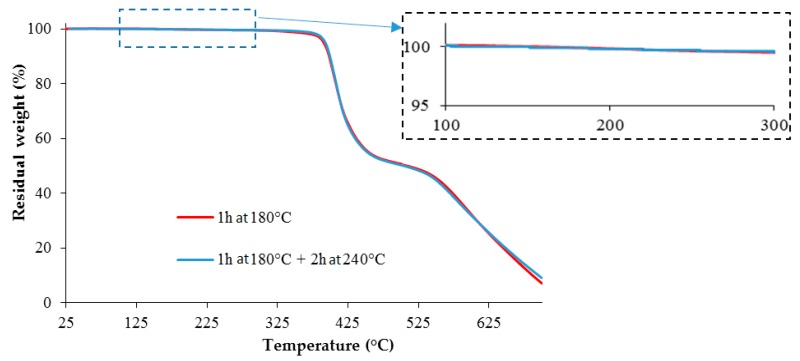
Comparison of two TETM/DDS samples cured under different curing cycles, by TGA, at a heating rate of 10 °C/min, under air.

Moreover, in order to confirm that the TETM/DDS formulations do not start to degrade during the post-cure at 240 °C, TGA analyses were performed on two samples (Figure 22). One sample has been subjected to the first stage at 180 °C only, and the second one has been subjected to the post-cure at 240 °C. We can observe that these curves are similar, which confirms that the post-cure does not initiate the degradation of the epoxy resin. The experimental result corroborates the fact that the increase in the thermogram (Figure 18) of an epoxy formulation with microspheres at 230 °C is only related to the degradation of the microspheres.

## 4. Conclusions

This paper reports on a complete study leading to the manufacture of a high temperature epoxy foam. The analyses performed on the expandable microspheres highlighted the different steps leading to the expansion. The copolymer shell passes through two main glass transition temperatures: the first one offers initial softening to reduce local pressure in the inner part of cell wall, whereas the second one leads to the hydrocarbons’ vaporization and the cell expansion. It was demonstrated that the expansion kinetics performed by TMA is even more important at higher temperatures. These results were correlated to the gel time of the epoxy formulation. Indeed, in order to maintain the bubble size and a homogeneous size distribution, gelation and foaming times have to be close. A good compromise temperature is 180 °C, because it allies great expansion with slow shrinkage and avoids thermal degradation.

In the second part of this paper, the TETM/DDS formulation curing process was optimized and a *T*_α_ of 330 ± 2 °C was obtained after a two-step curing cycle. Finally, the epoxy foam was manufactured and its curing process was optimized, leading to a *T*_α_ of 270 ± 2 °C.

## Figures and Tables

**Table 1:** Comparison of gel times for two mixing temperatures.

Mixing temperature	Gel time (min)						
	130 °C	140 °C	150 °C	160 °C	170 °C	180 °C	190 °C
100 °C	65.6 ± 4	46.3 ± 3.5	33.8 ± 2.2	18.3 ± 1	10.3 ± 1	6.1 ± 0.9	3.3 ± 0.8
180 °C	58.6 ± 4	41 ± 3.3	24.3 ± 2	13.6 ± 1	8.6 ± 1	5.0 ± 0.8	3.0 ± 0.8

**Table 2:** Density of foam samples.

Temperature	160 °C	170 °C	180 °C	190 °C	200 °C
Density (kg·m^−3^)	909 ± 14	797 ± 22	676 ± 27	495 ± 38	462 ± 42

## Abbreviations

The following abbreviations are used in this manuscript:

TETM	Triglycidyl ether of tris(4-hydroxyphenyl)methane
DDS	Diaminodiphenylsulfone
DSC	Differential scanning calorimetry
TGA	Thermogravimetric analysis
TMA	Thermomecanical analysis
SEM	Scanning electronic microscopy
